# Modulation of inflammatory response by electromagnetic field in Neuronal and Microglial cells

**DOI:** 10.26502/jsr.10020453

**Published:** 2025-07-02

**Authors:** Yssel Mendoza-Mari, Marija Stojanovic, Dan E Miulli, Devendra K Agrawal

**Affiliations:** 1 Department of Translational Research, College of Osteopathic Medicine of the Pacific, Western University of Health Sciences, Pomona CA 91766, USA; 2 Department of Neurosurgery, Riverside University Health System Medical Center, Moreno Valley, CA, USA

**Keywords:** Electromagnetic field, HMC3 cells, HCN2 cells, IL-1β, Microglial cells, Neurons, TNF-α, Traumatic Brain Injury

## Abstract

Neuroinflammation plays a key role in the development of CNS pathologies. This event encompasses a series of mechanisms involving the immune system and its cellular and molecular components. While it is necessary to activate the innate immune system during the early response to pathogens or traumas, persistent inflammation hinders neuronal recovery and contributes to the development of long-term neuronal complications. In this way, the application of pharmacological and non-pharmacological treatments is crucial to achieving better recovery of patients. We recently observed that the application of a low frequency electromagnetic field (EMF) decreases the expression of pro-inflammatory markers in an animal model of Traumatic Brain Injury in swine. To characterize this effect in terms of individualized response of neurons and microglial cells, we performed an in vitro model of pro-inflammatory damage by treating two different cell lines with tumor necrosis factor-α and then stimulating the cells with two frequencies of EMF. Transcriptional expression of inflammatory mediators was analyzed 24 and 48 hours after. Our results showed that both cell lines are susceptible to EMF, responding to the treatment by reducing the levels of the target genes in study. These observations further support the anti-inflammatory effect of EMF in the function of neurons and microglial cells and thus enhancing the recovery following traumatic brain injury, as observed under in vivo conditions in both experimental animals and human. These findings lay the foundation and warrants further preclinical and clinical studies to determine the effective frequency and duration of EMF stimulation in the healing of brain injury.

## Introduction

Activation of inflammasomes is a common feature of different neurological pathologies. These multimeric protein complexes are composed of three core elements: a sensor molecule, an effector enzyme, and an adaptor protein, which are expressed and acquire their structure in response to different danger signals such as pathogen-associated molecular (PAMPs) and damaged-associated molecular pattern molecules (DAMPs). In particular, the role of nucleotide-binding oligomerization domain leucine rich repeat and pyrin domain-containing protein 3 (NLRP3) inflammasome has been extensively characterized in several acute and chronic brain diseases as an important component of the neuroinflammatory process. After a “priming” stimulus, the expression of inflammasome components is increased. Afterwards, a secondary “activating” stimulus promotes the complex oligomerization in which NLRP3, apoptosis-associated speck-like protein (ASC), and caspase-1 associate to form the active structure [1]. Active caspase-1 cleaves pro-interleukin 1β (pro-IL-1β) and pro-IL-18 into their active isoforms, contributing to the establishment of a pro-inflammatory environment [2].

One of the “priming” stimuli for NLRP3 inflammasome is the binding of tumor necrosis factor alpha (TNF-α) to its receptor. This interaction switches on the transcription factor nuclear factor kappa-light-chain-enhancer of activated B cells (NF-κB), which relocates to the nucleus and upregulates the expression of NLRP3 and pro-IL-1β [3]. TNF-α also promotes cell necrosis, which leads to membrane disruption, DAMPs release, and necrosis, perpetuating a vicious circle of inflammation and cell death [4].

Among the different cell types present in the brain, microglia stand out for their key role in immunological surveillance and as mediators of the neuroinflammatory response [5]. The activation of these cells induces several pathways resulting in the release of pro-inflammatory cytokines such as IL-1β, IL-6 and TNF-α. Although crucial for the initial resolving of intrinsic and extrinsic damage signals, persistent inflammation contributes to the development of neurological complications and cell death [6]. It has been described that microglial cells actively express NLRP3 components [7] and also, with certain levels of discrepancy, oligodendrocytes, astrocytes [7–9] and neurons [10].

Electromagnetic field (EMF) application has become a very plausible non-pharmacological option for the treatment of neurological adquired malignancies and external damages as traumatic brain injury (TBI) [11–13]. Over the past 3 years, our group has studied the effects of low-frequency electromagnetic field (EMF) as a therapeutic option in a swine model of TBI based on controlled cortical impact (CCI) [14–16]. According to our observations, EMF exposure is associated with myelination, reactive oxygen species regulation, thyroid hormone transportation, cell proliferation, and cell migration, all of them contributing to increase the repairing process after trauma [17,18]. In particular, we observed a reduction in translational expression and protein content of NLRP3 inflammasome components, as well as pro-inflammatory mediators, in injured cortical areas of the brain. It was also shown that immediate application of EMF (20 minutes after TBI) was more effective in resolving inflammation than delayed treatment (two days post-TBI) [19].

To complement these observations, in the present study we evaluated the specific effect of EMF on the two major cellular types in the brain. We performed an in vitro model of inflammation by incubating the human cell lines HCN2 (cortical neurons) and HMC-3 (microglia) with recombinant human TNF-α. To evaluate the effect of EMF on the inflammasome components and pro-inflammatory cytokines, the cells were exposed to two different EMF frequencies, 20 minutes after the challenge with TNF-α. Transcriptional expression of target genes was analyzed 24 and 48 hours after EMF application.

## Materials and Methods

### Cell culture:

Human microglial cell line HCM3 (CRL-3304) ATCC, Manassas, VA, USA) and human cortical neuron cell line HCN-2 (CRL-3592) were purchased from ATCC (Manassas, VA, USA) and cultured according to the manufacturer’s recommendations. HMC3 cells were grown in Eagle’s Minimum Essential Medium (EMEM) (ATCC^®^ 30–2003) and HCN-2 cells in Dulbecco’s modified Eagle’s medium (DMEM), both supplemented with 10% fetal bovine serum (FBS) (Phoenix Research, Swedesboro, NJ, USA) and 1x antibiotic-antimycotic (penicillin, streptomycin, amphotericin B) (Gibco, Carlsbad, CA, USA) at 37°C and 5% CO_2_. During maintenance, the culture medium was replaced every 2 days. All experiments were carried out at cell passages < 10.

Pro-inflammatory treatment and electromagnetic field application: HMC3 and HCN2 cells were seeded in six-well plates at a density of 6×105 cells/well, 24 h prior to any treatment to ensure adhesion. Fresh culture medium supplemented with TNF-α (50 ng/mL) (300–01A-50UG, ThermoFisher Scientific, Waltham, MA, USA) was added to the cells, untreated cells were used as control. After 20 min, the plates were placed under a helmet provided with induction sensors (model BS-1000, Quasar Federal Systems, San Diego, CA) and dual-layered Mu-metal (MuMETAL^®^, Magnetic Shield Corporation, Bensenville, IL) previously described [14–16]. Stimulation thresholds of 2.5 or 5 Hz with 1 V signal intensity were applied during 3 min. After this time, plates were returned to their normal growing conditions of 37°C and 5% CO2. Cells not receiving EMF were considered as control.

Quantitative Real-Time Polymerase Chain Reaction (RT-qPCR): Semi-quantitative PCR analysis was performed at 24 and 48 h after the addition of treatments. Cell lysis was performed using TRIZOL (T9424, Millipore Sigma, Burlington, MA, USA) following the manufacturer’s instruction protocol in our laboratory. RNA pellets were resuspended in 30 μL of nuclease-free water (BP561–1, ThermoFisher Scientific, Waltham, MA, USA) and RNA yield was quantified using Nanodrop 2000 Spectrophotometer (Thermo Fisher, Waltham, MA, USA). Two micrograms of total RNA were used to synthesize complementary DNA (cDNA) using AzuraQuant^™^ cDNA Synthesis Kit (AZ-1996, Azura Genomics Inc., Raynham, MA, USA) according to manufacturer’s instruction using a T100^™^ Thermal Cycler (Bio-Rad Laboratories, Hercules, CA, USA). The cDNAs were diluted 1:20 in nuclease-free water and qPCR reactions were prepared in a final volume of 10 μL and in triplicate using AzuraView^™^ GreenFast qPCR Blue Mix LR (AZ-2350, Azura Genomics Inc., Raynham, MA, USA). Amplification was carried out in a C1000^™^ Thermal Cycler (Bio-Rad Laboratories, Hercules, CA, USA) and the cycling conditions were the following: 3 minutes at 95°C for initial denaturation, 40 cycles of 10 sec at 95°C (denaturation), 30 sec at 60°C (annealing/extension) followed by melting curve analysis. The primers for genes of interest and the housekeeping (Table 1) were purchased from Integrated DNA Technologies (Coralville, IA, USA). After normalization with 18S, relative gene expression was calculated using 2^−ΔΔCT^ method.

### Statistical analysis

Data were analyzed using GraphPad Prism 10 for Windows (version 10.3.0) and are represented as mean ± standard deviation. The normality of data was verified by Shapiro Wilk’s test. Fold change values obtained for each treatment were compared to untreated cells using One-way ANOVA and Dunnet’s posthoc test. Differences among TNF-α treated cells (with or without EMF) were analyzed by One-way ANOVA and Tukey’s posthoc test. For all analysis a p-value < 0.05 was accepted as statistically significant. Differences were represented by *p <0.05, **p<0.01, ***p <0.001 and ****p <0.0001.

## Results and Discussion

NLRP3 inflammasome is one of the main elements of the inflammatory response in acute and chronic human brain diseases. It is not only involved in the development of neurocognitive complications, but its components also constitute important therapeutic targets and biomarkers of predictive value for the recovery of patients. The role of NLRP3 as part of the innate immunological response after TBI has been extensively characterized [4]. Specifically, during the secondary phase of TBI, neuroinflammation is closely related to microglial activation and the production of pro-inflammatory cytokines such as TNF-α, IL-6 and IL-1β [20]. The perpetuation of inflammation after the initial mechanical trauma is critical in diminishing the negative long-term consequences. Several compounds have shown promising anti-inflammatory effects in experimental models of TBI. However, these results have not been extrapolated to the clinical setting, leaving an open niche for other pharmacological and non-pharmacological therapies. In this sense, the application of low frequency EMF constitutes a non-invasive therapy to be considered, based on positive experimental and clinical results obtained so far [21,22]. In our laboratory, the EMF application on a TBI model in swine evidenced a reduction of NLRP3 components and inflammatory markers at the transcriptional and translational levels [19]. To further characterize this effect of EMF on specific cell populations, we designed a study using cortical neuronal and microglial cell lines. Although an in vitro model does not fully replicate the complex interactions and environment of a whole organism, it constitutes a valuable platform to obtain fast and reproducible results regarding mechanisms of action.

For developing our model, we used the human cell lines HCM3 and HCN-2. HMC3 microglial cells have been widely employed to study microglial activation, phenotypes and mechanisms of action of different stressors like interferon-γ (IFN-γ), lipopolysaccharide and, amyloid β peptide among others [23]. HCN-2 is a cortical neuron-derived cell line that has been used in different models of neuronal damage [24]. The pro-inflammatory milieu was induced by treating the cells with TNF-α at 50 ng/mL. We have previously observed that this concentration is biologically active as it increases transcriptional expression of M1/M2 phenotypic markers in microglial cells, without reducing cellular viability (submitted results). Cells were exposed to EMF 20 minutes after TNF-α was added, taking into account that immediate EMF exerted better results in vivo in terms of reduction of inflammatory markers [19].

**Figure 1:**
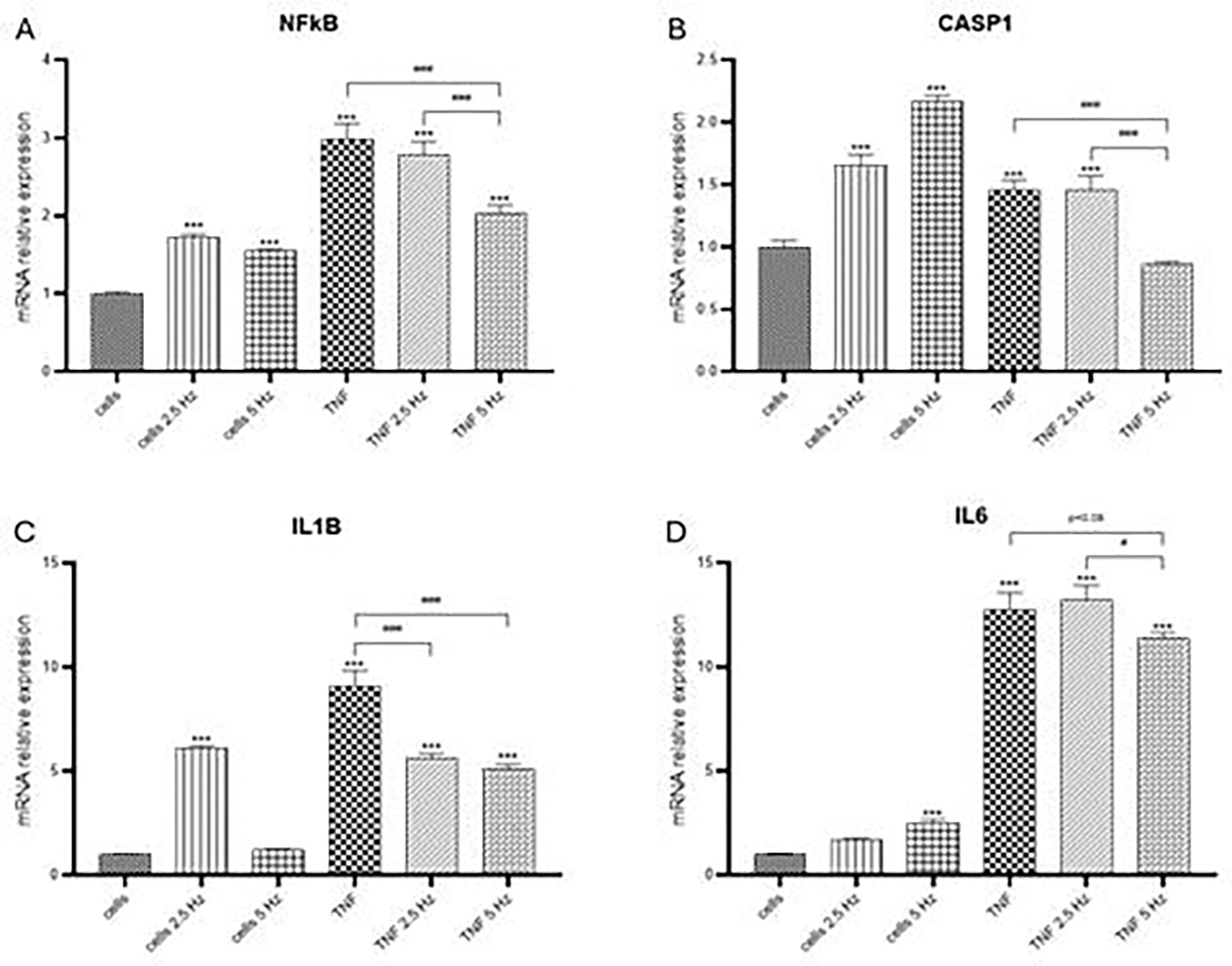
Effects of TNF-α and EMF exposition on the transcriptional expression of (A) NF-κB, (B) CASP1, (C) IL-1β, (D) IL-6 in HCN-2 cells. Cells were incubated with TNF-α (50 ng/mL) for 20 min and then exposed to EMFs of 2.5 or 5 Hz for 3 min. RT-PCR analysis were performed 24 h after the treatments. Data are presented as mean ± SD. Asterisks represent the statistical differences of each experimental condition compared to control untreated cells. Number signs represent the statistical differences among TNF-α treated cells. *(#) p<0.05, ** (##) p<0.01, *** (###) p<0.001. TNF-α: tumor necrosis factor alpha; EMF: electromagnetic field; NF-κB: nuclear factor kappa-light-chain-enhancer of activated B cells; CASP1: caspase 1; IL-1β: interleukin 1β; IL-6: interleukin 6.

TNF-α induced a significant increase in transcriptional expression of NF-κB, CASP1, IL-1β and IL-6 genes in the HCN-2 cortical cells (Figure 1). It has been previously described that TNF-α binding to its receptor activates a canonical signaling pathway that promotes the phosphorylation of NF-κB and its translocation to the nucleus, where it binds to the promoter regions of target genes, such as those encoding proinflammatory cytokines IL-6, IL-1β and TNF-α itself, stimulating their transcription [25]. Specifically, within neurons, NF-κB activation can produce both neuroprotective and neurodegenerative effects according to the specific context [26,27]. TNF-α can induce CASP1 expression, mediated by IRF-1 and p73, which activate the promoter through their respective binding sites [28]. TNF-α also induces CASP1 activation in an inflammasome-independent manner, through the ERK MAP kinase pathway [29]. In our experimental conditions, transcriptional expression of NLRP3 was not detected. This finding agrees with previous results in which NLRP3 was found primarily in microglia, but not in astrocytes nor neurons [30]. Nevertheless, there are some other studies in which NLRP3 was detected in the cerebral cortex of rats after TBI [10] or ischemic stroke [31]. In our study, we also could not detect IL-18 at transcriptional levels. This cytokine belongs to the IL-1 family of cytokine, and it has been defined as a proinflammatory cytokine with the ability to induce IFNγ [32]. The relationship between TNF-α and IL-18 expression in neurons is not well-established. While TNF-α is known to play a significant role in neuroinflammation and can influence the expression of various cytokines, most studies focus on its effects in glial cells, such as microglia and astrocytes, rather than neurons. Several repressors of IL-18 have been identified at the genomic and post transcriptional levels, including B cell lymphoma 6 protein and miRNAs [33]. Further studies are necessary to find possible explanations for our results.

When analyzing the effect of EMFs on the TNF-α induced pro-inflammatory markers we observed that the exposure to EMFs was associated with a significant reduction in translational expression of target genes (Figure 1). For NF-κB and CASP1 (Figure 1A, 1B), 5 Hz EMF showed a crystal-clear decrease in RNA levels compared to TNF-α treated cells; for IL6, this reduction was very close to significance, with a p value of 0.08 (Figure 1D). Frequency of 2.5 Hz only proved to be effective in reducing the levels of IL-1β (Figure 1C). In non-TNF-α treated cells we also observed that EMF exposure altered the transcriptional expression of genes in study, compared to no EMF control cells. It has been previously observed that pulsed electric fields alter the expression of NF-κB controlled genes in mammalian cell lines, increasing or decreasing the activity of their promoters [34]. Previous evidence suggests that different pulse duration and electric field strengths may promote diverse intracellular effects [35].

**Figure 2:**
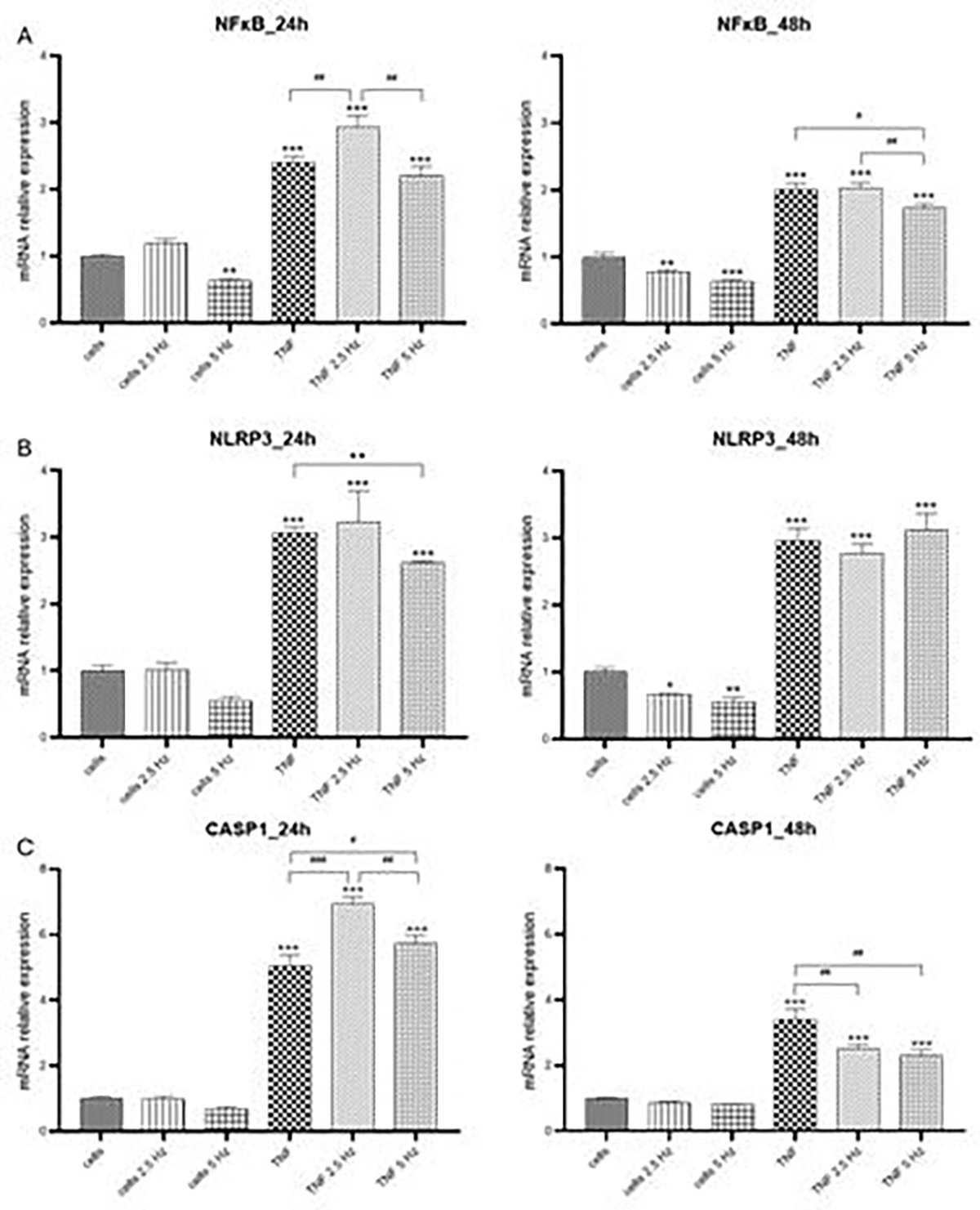
Effects of TNF-α and EMF exposition on the transcriptional expression of (A) NF-κB, (B) NLRP3, (C) CASP1 in HMC3 cells. Cells were incubated with TNF-α (50 ng/mL) for 20 min and then exposed to EMFs of 2.5 or 5 Hz for 3 min. RT-PCR analysis were performed 24 h and 48 h after the treatments. Data are presented as mean ± SD. Asterisks represent the statistical differences of each experimental condition compared to control untreated cells. Number signs represent the statistical differences among TNF-α treated cells. *(#) p<0.05, ** (##) p<0.01, *** (###) p<0.001. TNF-α: tumor necrosis factor alpha; EMF: electromagnetic field; NF-κB: nuclear factor kappa-light-chain-enhancer of activated B cells; NLRP3: nucleotide-binding oligomerization domain leucine rich repeat and pyrin domain-containing protein 3 CASP1: caspase 1.

In general, in HMC3 cells we observed some changes in transcriptional expression of genes of interest 24 h after the exposure to EMF, but the most evident effects were obtained 48 h after the addition of treatments. For NF-κB, 2.5 Hz frequency increased the expression of this transcriptional factor in cells treated with TNF-α, while 5 Hz had no effect. However, at 48 h, 5 Hz EMF significantly reduced NF-κB expression (Figure 2A), which could be important for further expression of NF-κB-dependent genes. For NLRP3 transcripts, there was an EMF-dependent reduction at 24 h when 5 Hz frequency was applied; there were no differences at 48 h (Figure 2B). CASP1 was highly induced in cells treated with TNF-α, both EMF frequencies significantly increased this level 24 h after exposure. Meanwhile, at 48 h there was a reduction in fold change values of CASP1 compared to 24 h; the application of EMF resulted in a significant decrease compared to TNF-α treated cells, with no differences between both frequencies applied (Figure 2C).

**Figure 3:**
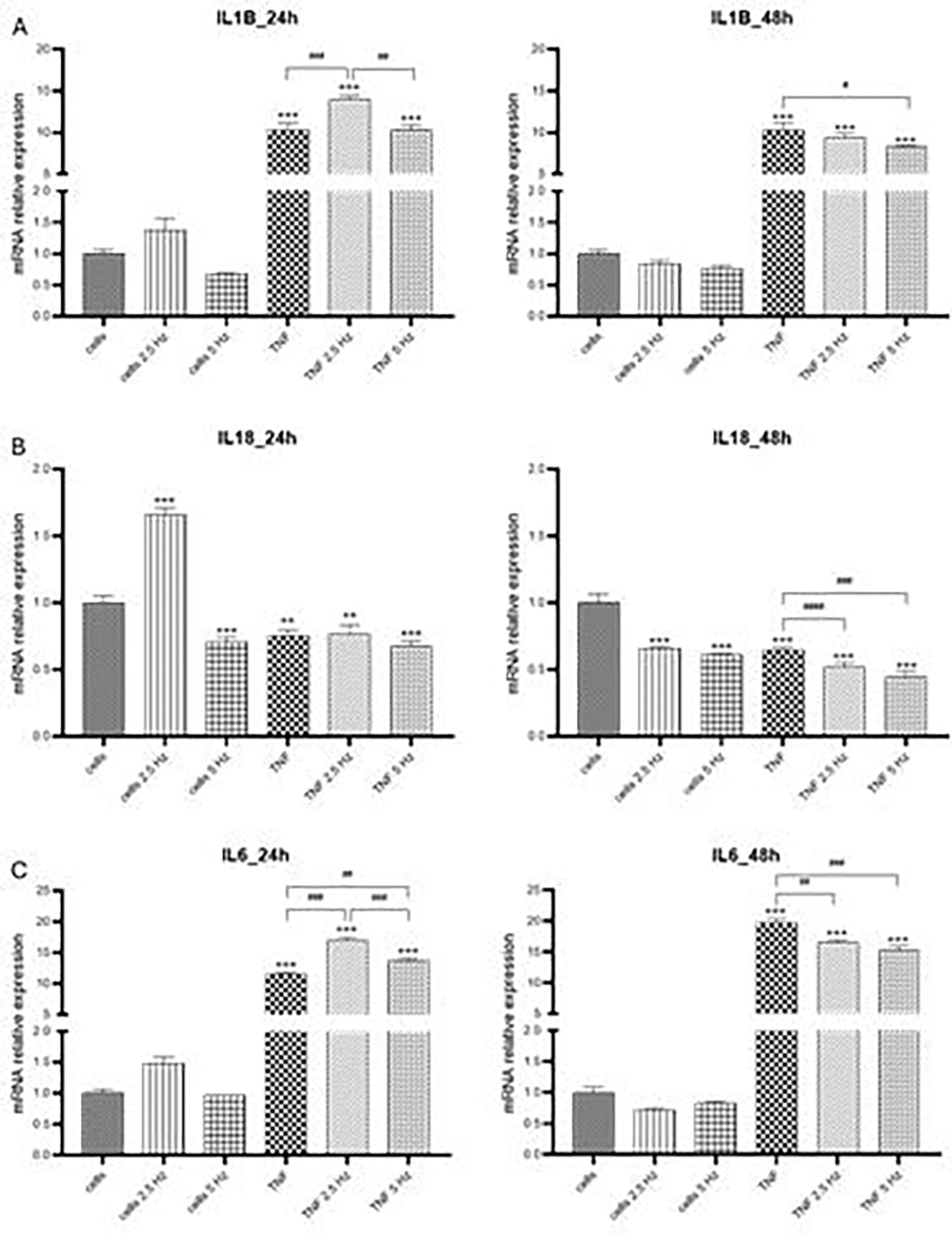
Effects of TNF-α and EMF exposition on the transcriptional expression of (A) IL-1β, (B) IL-18, (C) IL-6 in HMC3 cells. Cells were incubated with TNF-α (50 ng/mL) for 20 min and then exposed to EMFs of 2.5 or 5 Hz for 3 min. RT-PCR analysis were performed 24 h and 48 h after the treatments. Data are presented as mean ± SD. Asterisks represent the statistical differences of each experimental condition compared to control untreated cells. Number signs represent the statistical differences among TNF-α treated cells. *(#) p<0.05, ** (##) p<0.01, *** (###) p<0.001. TNF-α: tumor necrosis factor alpha; EMF: electromagnetic field; IL-1β: interleukin-1β; IL-18: interleukin 18; IL-6: interleukin 6.

Regarding pro-inflammatory cytokines we observed that TNF-α strongly induced the expression of IL-1β, and these levels remained the same at 24 and 48 h. Exposure to 2.5 Hz EMF significantly increased IL-1β after 24 h, similar to the effect observed in HCN2 cells. Five Hz EMF reduced IL-1β fold change after 48 h compared to TNF-α treated cells (Figure 3A). In our experimental system, transcriptional levels for IL-18 were not induced by TNF-α, on the contrary, they were significantly lower than that detected in the control cells (Figure 3B). EMF of 2.5 Hz significantly increased IL-18 at 24h, likewise it was observed for IL-1β in the two cell lines. Both EMF frequencies statistically reduced the expression of IL-18 at 48 h. The mRNA transcripts of expression of IL-18 is different to that detected for other cytokines that usually exhibit high levels of transcripts very early after a pro-inflammatory stimulus. For example, Liu et al. observed that IL-1β intensifies the inflammatory response in the early phase of TBI but IL-18 is involved in neuronal damage in the late phase post-TBI in a closed head weight drop TBI model in rats [10]. This is consistent with the results by Yatsiv et al. who detected increased IL-18 levels at 7 days following in a mouse model of closed head injury [36]. Further studies are needed to determine the pattern of expression of this cytokine over time. Finally, TNF-α highly induced the transcriptional expression of IL-6, in a time-dependent manner. At 24 h, exposure to both EMF frequencies increased IL-6 levels compared to cells that only received TNF-α. However, despite still showing high levels of transcriptional expression at 48 h, both EMF frequencies significantly reduced these values, compared to TNF-α treated cells. The results observed for NLRP3, IL-18 and IL-6 are in agreement with those we previously observed in vivo in which the immediate application of EMF after TBI reduced the transcriptional expression in the cortical tissue of the injured site [19].

## Conclusions

The results obtained in the present in vitro study allowed us to corroborate previous observations obtained in the pilot study of EMF application in a swine TBI model. The analysis allows us to conclude that both neuronal and glial cells, subjected to pro-inflammatory insults such as TNF treatment, respond to the application of an EMF with a decrease in the transcriptional expression of genes involved in the inflammatory response. Although with limitations, these results suggest that both cell types could be contributing in vivo to the anti-inflammatory response exerted by EMF. Furthermore, we were able to verify that the application of a 5 Hz frequency is more effective than that of 2.5 Hz, an element that should be considered and evaluated during future preclinical and clinical trials. Overall, this study contributes to expanding the research results on the potential application of EMF in CNS pathologies in which neuroinflammation plays a central role.

## Figures and Tables

**Table 1:** Sequences of forward and reverse oligonucleotides used for phenotypic characterization by RT-qPCR. 18S gene was used as a housekeeping gene to normalize results.

Phenotype marker	Forward	Reverse
NF-κB	5’- CTCCACAAGGCAGCAAATAGA-3’	5’- ACTGGTCAGAGACTCGGTAAA-3’
NLRP3	5’- TCCTCGGTACTCAGCACTAAT -3’	5’- AAGAGTCCCTCACAGAGTAGTT -3’
CASP1	5’- CTGCTCTTCCACACCAGATAAT-3’	5’- TTTCCTCCACATCACAGGAAC -3’
IL-1b	5’- GGTGTTCTCCATGTCCTTTGTA -3’	5’- GCTGTAGAGTGGGCTTATCATC-3’
IL-18	5’- CAACTCTCTCCTGTGAGAACAA-3’	5’- TTATCATGTCCTGGGACACTTC -3’
IL-6	5’- GGAGACTTGCCTGGTGAAA-3’	5’- CTCTGGCTTGTTCCTCACTAC-3’
18S	5’-CCCACGGAATCGAGAAAGAG-3’	5’-TTGACGGAAGGGCACCA-3’

CASP1: caspase 1; IL-1β: Interleukin 1β; IL-6: Interleukin 6; IL-18: Interleukin 18; NF-κB: nuclear factor kappa-light-chain-enhancer of activated B cells; NLRP3: nucleotide-binding oligomerization domain leucine rich repeat and pyrin domain-containing protein 3;.
